# Detection of Alzheimer's Disease using cortical diffusion tensor imaging

**DOI:** 10.1002/hbm.25271

**Published:** 2020-11-11

**Authors:** Mario Torso, Marco Bozzali, Giovanna Zamboni, Mark Jenkinson, Steven A. Chance, for the Alzheimers Disease Neuroimage Initiative

**Affiliations:** ^1^ Nuffield Department of Clinical Neurosciences University of Oxford Oxford UK; ^2^ Oxford Brain Diagnostics Oxford Centre for Innovation Oxford UK; ^3^ Neuroimaging Laboratory Santa Lucia Foundation Rome Italy; ^4^ Clinical Imaging Sciences Centre, Department of Neuroscience University of Sussex, Brighton & Sussex Medical School Falmer UK; ^5^ Dipartimento di Scienze Biomediche, Metaboliche e Neuroscienze Università di Modena e Reggio Emilia Reggio Emilia Italy; ^6^ Wellcome Centre for Integrative Neuroimaging, FMRIB, Nuffield Department of Clinical Neurosciences University of Oxford Oxford UK

**Keywords:** Alzheimer's Disease, cortical diffusion tensor imaging, cortical microstructure, diagnostic accuracy, minicolumn

## Abstract

The aim of this research was to test a novel in‐vivo brain MRI analysis method that could be used in clinical cohorts to investigate cortical architecture changes in patients with Alzheimer's Disease (AD). Three cohorts of patients with probable AD and healthy volunteers were used to assess the results of the method. The first group was used as the “Discovery” cohort, the second as the “Test” cohort and the last “ATN” (Amyloid, Tau, Neurodegeneration) cohort was used to test the method in an ADNI 3 cohort, comparing to amyloid and Tau PET. The method can detect altered quality of cortical grey matter in AD patients, providing an additional tool to assess AD, distinguishing between these and healthy controls with an accuracy range between good and excellent. These new measurements could be used within the “ATN” framework as an index of cortical microstructure quality and a marker of Neurodegeneration. Further development may aid diagnosis, patient selection, and quantification of the “Neurodegeneration” component in response to therapies in clinical trials.

## Graphical abstract

The aim of this research was to test a novel in‐vivo brain MRI analysis method that could be used in clinical cohorts, to investigate cortical architecture changes in patients with Alzheimer's Disease (AD).The new measures could be used within the “ATN” framework as an index of cortical microstructure quality and a marker of Neurodegeneration. Further development may aid diagnosis, patient selection, and quantification of the “Neurodegeneration” component in response to therapies in clinical trials.

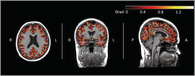

**Abbreviations**
AD: Alzheimer's Disease
AngleR: angle between the principal diffusion direction and the minicolumn direction within the cortex
GM: grey matter
MD: mean diffusivity
ParlPD: the component of the principal diffusion vector that was parallel to the minicolumn direction within the cortex
PerpPD: the component of the principal diffusion vector perpendicular to the minicolumn direction within the cortex

## 1 INTRODUCTION

Approximately 50% of dementia sufferers are thought to be undiagnosed, particularly in the early stages of disease. Early detection of Alzheimer's Disease (AD) also presents a challenge for patient inclusion in drug trials, which may have contributed to trial failures costing pharmaceutical companies billions of dollars. New ways to quantify AD are needed to add to the repertoire of existing methods if we are to overcome these challenges. The newly proposed Alzheimer's classification framework suggests a description based on a patient's biomarker profile (Jack Jr et al., 2018). In this “ATN” framework (“A” for amyloid deposition, “T” for tau levels, and “N” for neurodegeneration) AD forms a continuum in which the extreme points are represented by A−T−N−cognitively unimpaired subjects, and A+ T+ N+ subjects with dementia. The present study focused on change in the underlying neural architecture responsible for cognitive function as a potential “N” biomarker.

In addition to cell loss and synapse loss, the vertical cellular micro‐circuits, known as minicolumns, which constitute the fundamental structure throughout the cerebral cortex, are altered in a graded manner during ageing, mild cognitive impairment (MCI), and AD (Chance et al., 2011). The microscopic disruption of columnar architecture correlates with plaque load and cognitive decline (van Veluw et al., 2012). A novel analysis method using Diffusion Tensor MRI to measure correlates of these cortical microstructural changes was previously validated against postmortem histology (McKavanagh et al., 2019) and tested in in‐vivo cohorts (Dickstein et al., 2020; Torso, Ahmed, et al., 2020; Torso, Bozzali, et al., 2020).

The present study aimed to provide the first preliminary in‐vivo validation of these neuroimaging measurements in AD cohorts to demonstrate that they are sensitive to dementia‐related microstructural changes. This analysis method is complementary to other “N” biomarkers and requires only conventional MRI scanners, standard diffusion protocols, and no contrast agents. It is, therefore, potentially applicable to a variety of acquisition environments, including clinical.

This study aimed to test: (1) if the cortical diffusivity analysis provided generalizable in vivo measures of cortical grey matter diffusivity; (2) if the cortical diffusivity analysis can discriminate between groups; (3) how the discriminative power of the method compared with other clinical biomarkers (Cortical grey matter volume, AV45, and AV1451 PET for amyloid and tau).

## 2 METHODS

### 2.1 Study participants

A total of 78 individuals with probable AD and 71 healthy elderly controls (HC) from three different cohorts were included in the study.

The first cohort (24 AD, 23 HC) was an existing dataset recruited in Oxford (UK) (Zamboni et al., 2013) and was used as a “Discovery cohort” to explore the in‐vivo validity of a novel method of cortical diffusivity analysis (Table 1).

**TABLE 1:** Demographic and clinical characteristics

Dataset	Diagnosis	Age (years)	Sex (F/M) (%)	Education (years)	MMSE score	CDR	Apoe4 carrier (%)	Hachinski score
Discovery cohort (Oxford)	HC *n* = 23	75.4 ± 8.34	45/55	15.6 ± 3.25	29.4 ± 0.88#	0 ± 0#	35	0.46 ± 0.28
	AD *n* = 24	73.5 ± 6.43^a^	40/60^b^	13.4 ± 3.39^a^	22.4 ± 3.33^a^	0.90 ± 0.34^a^	55^b^	0.81 ± 0.62^a^
Test cohort (Rome)	HC *n* = 23	71.23 ± 6.23	53/47	13.0 ± 2.82	29.1 ± 1.52#	0 ± 0#	‐	0.38 ± 0.41
	AD *n* = 29	72.37 ± 4.43^a^	60/40^b^	10.2 ± 3.76^a^	19.9 ± 4.05^a^	1.06 ± 0.29^a^	‐	0.72 ± 0.55^a^
ATN cohort (ADNI 3)	HC *n* = 25	74.35 ± 10.04	56/44	16.2 ± 2.17	29.2 ± 0.81#	0 ± 0#	42	0.26 ± 0.16
	AD *n* = 25	74.30 ± 10.08^a^	56/44^b^	15.2 ± 2.11^a^	20.4 ± 3.47^a^	1.02 ± 0.53^a^	64^b^	0.20 ± 0.40^a^

*Note:* For each group of subjects, the table shows the mean (SD) of age, years of formal education, MMSE, CDR scores and percentages of sex distribution. *p* < .05 after Bonferroni's Correction for multiple comparisons (statistical threshold = *p* < .006 [0.05/8]). “#” denotes significant difference.

Abbreviations: AD, Alzheimer's Disease; CDR, Clinical Dementia Rating scale; HC, healthy controls; MMSE, Mini Mental State Examination.

^a^ *t*‐test.

^b^ Chi‐square.

The second cohort (29 AD, 23 HC) was an existing dataset recruited in Rome (Italy) (Giulietti et al., 2018) and was used as a “Test cohort” to test repeatability of the method in an independent sample (Table 1).

All subjects underwent extended clinical and neuropsychological assessments, which were centre specific (Giulietti et al., 2018; Zamboni et al., 2013), but included the Mini Mental State Examination (MMSE) (Folstein, Robins, & Helzer, 1983) and the Clinical Dementia Rating scale (CDR) (Hughes, Berg, Danziger, Coben, & Martin, 1982). Inclusion criteria for healthy elderly controls were: MMSE score between 24 and 30, a CDR of 0, no psychopharmacological treatment, no subjective memory complaints, absence of psychiatric and neurological conditions, absence of history of cancer, non‐MCI, and non‐demented.

A third cohort (25 AD, 25 HC) was selected from the Alzheimer's Disease Neuroimaging Initiative 3 database (ADNI 3). This is the most up‐to‐date ADNI cohort with a consistent acquisition protocol. All AD subjects available at the time of the study design were included if they had biomarker data enabling them to be classified according to the ATN framework (Jack Jr et al., 2018) and had MRI data acquired using consistent ADNI3 protocols. A matched HC group was selected. All subjects of this cohort were characterized based on the ATN framework, using UC Berkeley AV45 Florbetapir and AV1451 Flortaucipir PET values. The AV45 PET standard uptake value ratio (SUVR) (whole cerebellum reference region) values were used to assess amyloid deposition: T1 scans for each subject were segmented and parcellated with Freesurfer to define cortical grey matter regions of interest (frontal, anterior/posterior cingulate, lateral parietal, and lateral temporal) that make up a summary cortical ROI. The AV45 Florbetapir SUVR values were calculated by averaging across the four cortical regions and dividing this cortical summary ROI by the whole cerebellum reference region (cutoff of 1.11; Joshi et al., 2012). TheAV1451 PET SUVR values were used to assess tau lesions. The SUVR values were calculated by dividing the region of interest (Braak stage V composite value) by a reference region (cerebellar GM, cutoff 1.33; Jack Jr et al., 2017).

The clinical diagnosis was given according to the National Institute of Neurological and Communicative Disorders and Stroke‐Alzheimer''s Disease and Related Disorders Association (NINCDS‐ADRDA) criteria (McKhann et al., 2011). This cohort was used as the “ATN” cohort to test the validity of the novel cortical diffusion metrics and their potential role as “N” biomarkers compared with other “A” and “T” biomarkers. To better describe the cohort, APOE genotype was added. Note that, the tests of diagnostic accuracy for the ATN cohort (below) used the subject clinical diagnosis as the criteria for diagnostic grouping. The ATN markers were only used as a framework for reference, but did not form part of subject categorization for classification testing within this study.

### 2.2 Structural MRI analyses

All participants had undergone MRI scanning to acquire T1 Structural and Diffusion weighted scans.

The 3D T1‐weighted image was segmented using FreeSurfer v6.0 (http://surfer.nmr.mgh.harvard.edu/) to compute GM fraction (GM fr), Bilateral Hippocampal fraction (Hipp Bil fr), and White Matter Hypointensities fraction (WMHs fr) (see Supplementary material).

### 2.3 DTI analysis

DTI preprocessing was performed using FSL tools (http://www.fmrib.ox.ac.uk/fsl/) (see Supporting Information).

To control for the effect of head motion (Baum et al., 2018) in DTI maps, a displacement index generated using an in‐house script was calculated (see Supporting Information). This value was used as a covariate in the General Linear Model (GLM) multivariate analysis.

### 2.4 Cortical diffusivity analysis

The automatic cortical diffusivity analysis consisted of several different stages performed using a proprietary software tool (see Supporting Information and McKavanagh et al., 2019). In summary, the tool generates cortical profiles, that is, lines estimating the columnar axis within the cerebral cortex. Values for the diffusion tensor derived metrics were averaged along the cortical profiles, across the whole cortical grey matter to provide a global, summary value for each one. The metrics calculated were mean diffusivity (MD) and three measures relating to the principal diffusion component, namely: the angle between the radial minicolumn direction within the cortical GM and the principal diffusion direction (AngleR, *θ*rad); the principal diffusion component projected onto the plane perpendicular to the radial minicolumn direction (PerpPD, *D*1,⊥ [×10^−3^ mm^2^/s]), and the principal diffusion component projected onto, and therefore parallel with, the radial minicolumn direction (ParlPD, *D*1,∥ [×10^−3^ mm^2^/s]).

Readers may be familiar with MD as a measure of the total diffusion occurring in a voxel. It is calculated by finding an average of the three eigenvalues (i.e., [L1 + L2 + L3]/3). In the present study, additional measures were calculated as described in US20180143282A1: The perpendicular diffusivity was determined by multiplying the main eigenvector (V1) by the value of its corresponding eigenvalue (L1), then resolving this into its components. The value of the component perpendicular to the radial minicolumn direction across the cortex was the perpendicular diffusivity. Radial or parallel diffusivity was the component of the diffusion occurring in the principal diffusion direction that was parallel to the radial minicolumn direction across the cortex. The angle of columnar deviation, also called AngleR, was the difference between the radial minicolumn direction across the cortex, and the direction of the main eigenvector (V1), expressed as an angle.

The direction, CRadial, was derived, spanning the cortical ribbon between the pial and white matter boundary surfaces. Over 100,000 approximately evenly‐spaced points on the white matter surface were taken, and cortical profiles were propagated through the cortical layers replicating the histological principles of radial minicolumns (Rakic, 1995), aiming to minimize the crossings of profiles, and reflecting the inside‐out migration of cells along radial glial guidelines toward corresponding points representative of Cajal–Retzius cells at the pial surface. The cortical profiles were then selected for inclusion, taking into account features of cortical geometry that are known to influence or correlate with minicolumn width, shape and cell density, including cortical thickness and curvature.

All the cortical values were averaged to reduce the influence of noise in the DTI scans, effectively smoothing the data, and ensuring only directionality with some local coherence would dominate, therefore guarding against the influence of random deflections from the minicolumn direction. Each of the three novel metrics, AngleR, PerpPD, and ParlPD, was based on an average from the whole cortex. As with other widely used metrics, such as whole brain volume (which does not discriminate between the many tissue compartments and sub‐structures), a summary value for each subject has the advantage that it provides a good overview of group differences without the complications of sub‐region sampling, requiring multiple covariates and multiple testing corrections.

### 2.5 Validity

To test the validity of the method, the study design enabled the assessment of several different validity requirements:

- *Repeatability*: investigated as intrascanner variation, that is, the degree of variation produced by running the cortical diffusivity analysis on the same subjects (six controls) at two different time points, baseline and follow‐up after a three‐month interval, acquired on the same scanner.
- *Reliability*: investigated as Interscanner variation, that is the degree of variation produced by running the cortical diffusivity analysis on similar cohorts, acquired on different scanners. To do that, the cortical diffusivity analysis was run on the Discovery, Test and ADNI3 cohorts.
- *Construct validity*: the degree to which the cortical diffusivity analysis measured what it claimed to be measuring, was assessed using correlations between cortical diffusivity analysis measures and other common indices of brain structural degeneration (GM fr, Hipp bil fr, and WMHs fr) and global cognitive status (MMSE).
- *Concurrent validity*: the relationship between the measures obtained through the novel method and the standard disease measures included in the ATN framework (AV45 PET SUVR composite values and the AV1451 PET SUVR values), was assessed in the ATN cohort.

### 2.6 Diagnostic accuracy

To test the diagnostic accuracy of the cortical diffusivity analysis measures, different indices were estimated. The group discrimination capability (diagnostic group: HC vs. AD) of cortical diffusivity measures was investigated using Receiver Operating Characteristics (ROC) curve analysis and compared with a conventional diffusion measure (MD) and GM_fr (considered as a measure of atrophy and one of the main measures of neurodegeneration). As is well known, hippocampal atrophy is a sub‐region value and is one of the main criteria to define AD diagnosis and therefore formed part of the group classification criteria, so it was not used in the discrimination capability comparison.

We considered as the “best discriminator” the feature with the highest area under the ROC curve (AUC). Finally, to summarize the predictive value of each measurement, the accuracy (ACC), sensitivity (SENS), specificity (SPEC), positive likelihood ratio (LR+), negative likelihood ratio (LR−), Youden's J statistic, positive predictive value (PPV), and negative predictive value (NPV) were computed at the best point along the ROC curve for each measurement. We defined the best point as the one with the highest value obtained by averaging sensitivity and 1 − specificity.

### 2.7 Statistical analysis

Data were analyzed using IBM SPSS Statistics version 25 (SPSS, Chicago, IL).

The multivariate General Linear Model (GLM) of SPSS was used to compare between‐group differences in cortical diffusivity measures in the cohorts, using group membership as a fixed factor and head movement, subject age, and scanner as covariates. Differences between groups were tested with *χ* ^2^‐tests and *t*‐tests. All the statistical results were thresholded at *p* < .05, after Bonferroni correction (0.05/number of comparisons).

Pearson's and Spearman's correlations were used to investigate the associations among measurements. All p‐values in correlation analysis were adjusted with false discovery rate correction (FDR <0.05) (Benjamini & Yekutieli, 2001).

The intrascanner variation (T0–T1) of each cortical diffusivity measure was estimated using Cronbach's *α*. The intraclass correlation coefficient (ICC) and its 95% confidence interval (95% CI) were used to estimate reliability.

## 3 RESULTS

### 3.1 Demographics and clinical values

Table 1 summarizes the principal demographic and clinical characteristics of all subjects included in the study.

In all cohorts, no significant difference was observed between groups for age, years of formal education, or sex.

As expected, in all cohorts *t*‐tests revealed lower MMSE (*p* < .0001) and higher CDR score (*p* < .0001) in the AD groups.

### 3.2 Structural MRI analysis

Volumetric brain values are summarized in Table 2.

**TABLE 2:** Brain volumetrics

Dataset	Diagnosis	Grey matter fraction GM fr	Hippocampal fraction Hip fr	WMHs fraction WMHs fr
Discovery cohort (Oxford)	HC *n* = 23	0.265 ± 0.037#	0.00465 ± 0.00051#	0.00391 ± 0.0060
	AD *n* = 24	0.221 ± 0.024^a^	0.00340 ± 0.00072^a^	0.00517 ± 0.0057^a^
Test cohort (Rome)	HC *n* = 23	0.267 ± 0.035#	0.00470 ± 0.00081#	0.00248 ± 0.0015#
	AD *n* = 29	0.222 ± 0.025^a^	0.00320 ± 0.00079^a^	0.00409 ± 0.0024^a^
ATN cohort (ADNI 3)	HC *n* = 25	0.292 ± 0.018#	0.00502 ± 0.00060#	0.00220 ± 0.0024
	AD *n* = 25	0.267 ± 0.023^a^	0.00373 ± 0.00058^a^	0.00373 ± 0.0039^a^

*Note:* The table shows the mean (*SD*) of Grey Matter fraction. Hippocampal Bil fraction and WMHs fraction. *p* < .05 after Bonferroni's Correction for multiple comparisons (statistical threshold = *p* < .016 [0.05/3]). “#” denotes significant difference.

Abbreviations: AD, Alzheimer's Disease; HC, healthy controls.

^a^ *t*‐test.

In the Discovery cohort, AD patients showed a significantly lower GM fraction than HC (*t*_45_ = 4.457; *p* < .0001). As expected, the AD group showed a significantly lower Hipp Bil fr in (*t*_45_ = 4.695; *p* < .0001). No significant between‐group difference was found for the WMHs fr.

In the Test cohort, AD patients showed a significantly lower GM fraction (*t*_50_ = 5.831; *p* < .0001). and Hipp Bil fr (*t*_50_ = 6.934; *p* < .0001) than HC. Moreover, the t‐test analysis showed a significantly higher WMHs fr in the AD group (*t*_50_ = 5.253; *p* < .010).

In the ATN cohort, the AD group showed a significantly lower GM fraction (*t*_48_ = 7.872; *p* = .000.) and Hipp Bil fr (*t*_48_ = 7.691; *p* = .000) compared to the HC group.

### 3.3 Cortical DTI analysis—results and validity

The results of the repeatability test, based on the comparisons between baseline and the 3‐month follow‐up, revealed that all cortical diffusivity measures (AngleR, MD, PerpPD, and ParlPD) had good to excellent ICC (*α* = .89–.93).

Concerning reliability (interscanner variation), the differences between HC and AD groups were tested in each cohort:

In the Discovery cohort, the GLM showed that just the diagnosis had a significant overall effect (*F*_4,42_ = 11.048; *p* < .0001). No significant effects of head movement or age were detected. Between subjects, MD (*F*_1,46_ = 26.701; *p* < .0001), AngleR (*F*_1,46_ = 29.950; *p* < .0001), PerpPD (*F*_1,46_ = 25.483; *p* < .0001), and ParlPD (*F*_1,46_ = 16.014; *p* < .0001) were higher in AD.

In the Test cohort, the multivariate test showed a significant effect of diagnosis (*F*_4,45_ = 16.435; *p* < .0001). No significant effects of head movement or age were found. Between‐subject effects revealed that MD (*F*_1,51_ = 19.313; *p* < .0001), AngleR (*F*_1,51_ = 50.088; *p* < .0001) and PerpPD (*F*_1,51_ = 30.465; *p* < .0001) and ParlPD (*F*_1,51_ = 11.055; *p* < .001) were higher in AD.

Finally, in the ATN cohort HC and AD were compared, controlling for the effects of diagnosis, age, head movement and scanner. The results showed significant effects of group diagnosis (*F*_4,43_ = 8.551; *p* < .0001) and head movement (*F*_4,43_ = 16.992; *p* < .0001).

The tests of between‐subject effects revealed that AngleR (*F*_1,49_ = 29.658; *p* < .0001) and PerpPD (*F*_1,49_ = 8.419; *p* < .001) were higher in AD. There was also an effect of head movement on MD (*F*_1,49_ = 16.747; *p* < .0001), PerpPD (*F*_1,49_ = 8.678; *p* < .005), and ParlPD (*F*_1,49_ = 13.899; *p* < .001). No other effects were found. All of the results reported here remained significant after Bonferroni correction.

Construct validity was investigated using Pearson's correlation coefficient to test the relationships between cortical diffusivity measures, structural and clinical variables. Several significant correlations were found (see Table 3 for more details).

**TABLE 3:** Construct validity

Discovery cohort (Oxford)		MMSE	Hipp Bil fr	GM fr	WMHs fr	Hachinski score	CDR	ApoeE4#	AV45 SUVR	AV1451 SUVR
MD	*r* *p*	−.478* .002	−.404* .010	−.522* .001	.286 .090	.313* .032	.632* .000	—	—	—
AngleR	*r* *p*	−.582* .000	−.592* .000	−.610* .000	.301* .027	.466* .001	.605* .000	—	—	—
PerpPD	*r* *p*	−.541* .000	−.348* .028	−.709* .000	.404* .015	.332* .026	.638* .000	—	—	—
ParlPD	*r* *p*	−.503* .000	−.471* .001	−.710* .000	.273* .045	.276* .044	.577* .000	—	—	—

Test cohort (Rome)		MMSE	Hipp Bil fr	GM fr	WMHs fr	Hachinski score	CDR	ApoeE4#	AV45 SUVR	AV1451 SUVR
MD	*r* *p*	−.591* .000	−.566* .000	−.668* .000	.307* .030	.293* .037	.649* .000	.079 .610	—	—
AngleR	*r* *p*	−.532* .000	−.750* .000	−.705* .000	.322* .021	.428* .002	.671* .000	.108 .487	—	—
PerpPD	*r* *p*	−.611* .000	−.594* .000	−.719* .000	.325* .020	.307* .029	.703* .000	.093 .546	—	—
ParlPD	*r* *p*	−.529* .000	.413* .002	−.591* .000	.280* .049	.216 .128	.591* .000	.122 .428	—	—

ATN cohort (ADNI 3)		MMSE	Hipp Bil fr	GM fr	WMHs fr	Hachinski score	CDR	ApoeE4#	AV45 SUVR	AV1451 SUVR
MD	*r* *p*	−.502* .000	−.437* .002	−.387* .006	.110 .145	.405* .009	.473* .001	.044 .767	.212 .177	.283 .069
AngleR	*r* *p*	−.562* .000	−.599* .000	−.632* .000	.331* .019	.433* .002	.612* .000	.222 .134	.616* .000	.373* .015
PerpPD	*r* *p*	−.575* .000	−.549* .000	−.510* .000	.176 .221	.326* .031	.546* .000	.228 .152	.307 .048	.351* .023
ParlPD	*r* *p*	−.427* .002	−.397* .004	−.339* .016	.087 .549	.297 .040	.433* .002	.111 .457	.166 .294	.293 .062

*Note:* Pearson's correlation among studied measures. “#” denotes Spearman's correlation.

* Significant after false discovery rate correction (FDR <0.05).

**FIGURE 1:**
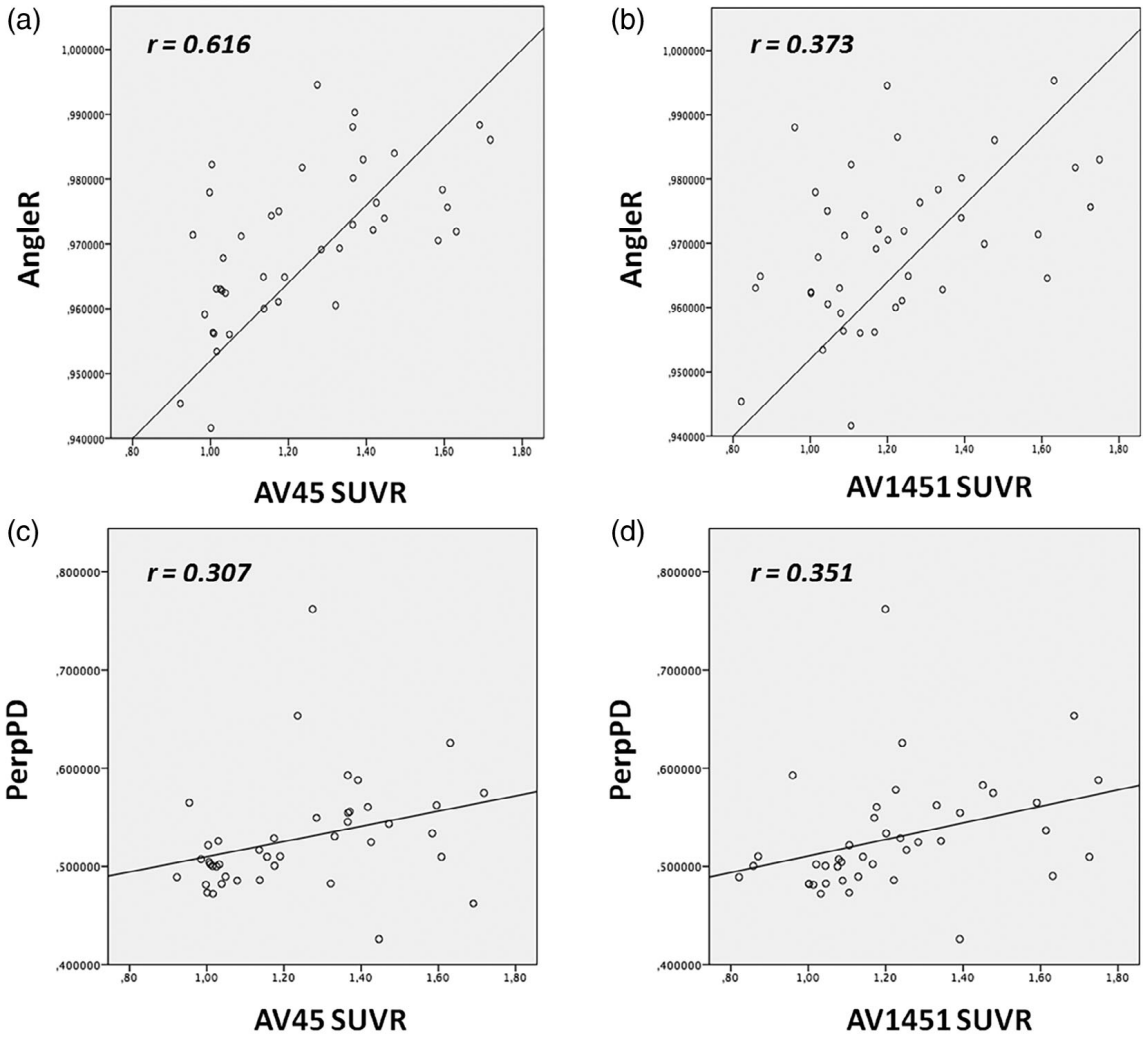
Correlations between cortical diffusion (AngleR and PerPD) and PET SUVR values (AV45 and AV1451). The graphs show significant positive correlations between a) AngleR and AV45 SUVR values (*r* = .616; *p* = .000, pFDR = 0), (b) AngleR and AV1451 SUVR (*r* = .373; *p* = .015, pFDR = .045), (d) PerpPD and AV1451 SUVR (*r* = .351; *p* = .023 pFDR = .046). The correlation between (c) PerpPD and AV45 SUVR values did not survive FDR correction (*r* = .307; *p* = .048, pFDR = .072)

Concerning the relationships among the cortical diffusivity analysis measures and the other biomarkers included in the ATN framework (concurrent validity), correlation analyses revealed significant positive correlations between AngleR and AV45 SUVR values (*r* = .616; *p* = .000, pFDR = 0), AngleR and AV1451 SUVR (*r* = .373; *p* = .015, pFDR = .045) PerpPD and AV1451 SUVR (*r* = .351; *p* = .023 pFDR = .046). The correlation between PerpPD and AV45 SUVR values did not survive FDR correction (*r* = .307; *p* = .048, pFDR = .072). No significant correlations between ParlPD and PET values were found (Figure 1).

### 3.4 Diagnostic accuracy (classification effectiveness in comparison to other methods)

**FIGURE 2:**
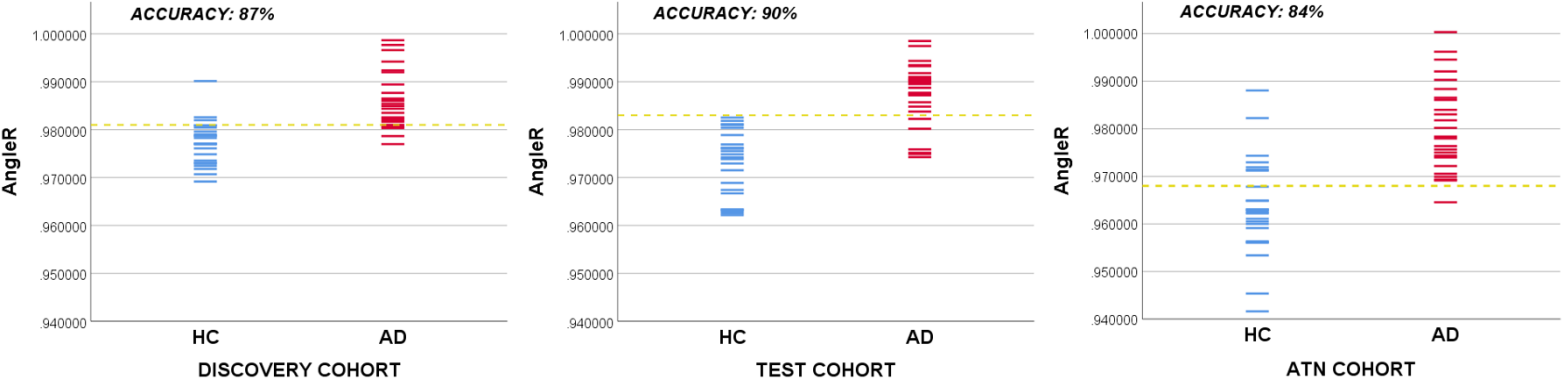
Discriminations using the AngleR cutoff corresponding to the “best” point in the ROC curves

The ability of each measure to correctly classify AD and non‐AD subjects was assessed using the receiver operating characteristics (ROC) curve. Table 4 and Figure 2 show the principal measures of diagnostic reliability of cortical diffusivity measures. For each group, ROC analysis was performed on the structural volume measure (GM fr) and diffusion cortical indices (MD, AngleR, PerpPD, and ParlPD).

**TABLE 4:** Diagnostic indices

	AUC	95% CI	CUT‐OFF	SENS (%)	SPEC (%)	ACC (%)	PPV (%)	NPV (%)	J	LR−	LR+
**Discovery cohort (Oxford)**											
MD	0.862	0.747–0.963	1.0979	79	83	81	83	79	0.617	0.25	4.5
AngleR	**0.887**	0.791–0.983	0.98096	88	87	**87**	88	87	0.744	0.14	6.7
PerpPD	0.850	0.733–0.967	0.63542	71	91	81	89	75	0.621	0.31	8.1
ParlPD	0.792	0.655–0.930	0.41828	63	91	77	88	70	0.538	0.41	7.2
GM fr	0.816	0.695–0.937	0.27642	67	91	79	89	72	0.579	0.36	7.6
**Test cohort (Rome)**											
MD	0.858	0.750–0.965	1.09798	90	78	85	84	86	0.679	0.13	4.12
AngleR	**0.931**	0.866–0.997	0.98313	86	96	**90**	96	85	0.815	0.14	20.6
PerpPD	0.903	0.812–0.993	0.65594	81	92	87	91	83	0.730	0.21	10.5
ParlPD	0.798	0.668–0.928	0.43162	90	65	79	76	83	0.548	0.16	2.6
GM fr	0.860	0.772–0.966	0.24313	86	74	81	81	81	0.601	0.19	3.3
**ATN cohort (ADNI 3)**											
MD	0.629	0.461–0.797	0.95613	68	80	74	77	71	0.48	0.40	3.4
AngleR	**0.896**	0.808–0.984	0.96884	96	72	**84**	77	95	0.68	0.06	3.4
PerpPD	0.666	0.504–0.827	0.52954	64	84	74	80	70	0.48	0.43	4
ParlPD	0.597	0.428–0.766	0.36071	60	76	68	71	65	0.44	0.52	2.5
GM fr	0.798	0.651–0.945	0.27578	64	88	76	84	71	0.52	0.41	5.3
AV45 PET	—	—	1.11	88	64	74	64	88	0.58	0.30	4.5
AV1451 PET	—	—	1.33	55	96	78	91	74	0.51	0.46	13.33

*Note:* The Table shows the accuracy indices for each studied measure. The bold values represent the best diagnostic performance. AngleR values are expressed in radians (Θrad); MD, PerpPD, and ParlPD values are expressed in (×10^−3^ mm^2^/s).

Abbreviations: 95% CI, 95% confidence interval; ACC, Accuracy; AUC, area under the curve; GMfr, Grey matter fraction; J, Youden's J statistic; LR+, LR−, likelihood ratio positive and negative; MD, mean diffusivity; NPV, negative predictive values; PPV, positive predictive values; SENS, sensitivity; SPEC, specificity.

In the Discovery cohort, as expected, GM fr provided good group discrimination, having AUC = 0.816.

Among diffusion cortical values, AngleR was the best between‐group discriminator, having an AUC = 0.887, followed by MD (AUC = 0.862) and PerpPD (AUC = 0.850). The AngleR cutoff, designated the “best” point of the ROC curve was 0.981 *θ*rad.

**FIGURE 3:**
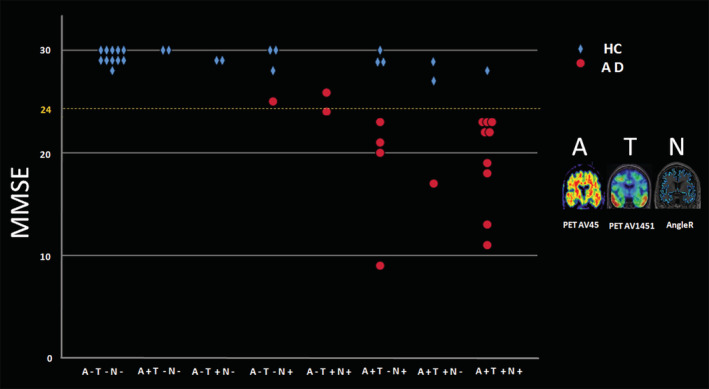
This figure shows that no single marker, including MMSE score, is adequate for identifying patients with a clinical diagnosis of Alzheimer's Disease. The *x* axis shows categories with increasing number of positive markers. In general, subjects with only one positive marker (toward the left side) are healthy controls (blue) and indicate false positives for the individual markers which report a positive. Whereas, with increasing combined marker positivity, more subjects are found to be AD patients (red) and on the right side of the graph the red points indicate false negatives for those markers which report a negative. Interestingly, a few AD patients were Amyloid negative subjects with above threshold MMSE, but were positive on the other markers. (Note that the ADNI3 protocol defined AD subjects with MMSE within the range 20–24, however, some subjects presented here were carried over from the earlier ADNI data sets within which the original criteria defined AD with MMSE 20–26)

Therefore, additional statistics were generated for AngleR: Likelihood ratio values (LR− = 0.14; LR+ = 6.7) revealing that individuals with AngleR values greater than 0.981 *θ*rad (Figure 3) had an increased probability of disease compared to individuals with lower AngleR values. Moreover, using the cut‐off of 0.981 *θ*rad all individuals were classified with an accuracy of 87% and a J of 0.74, NPV (87%) and PPV (88%).

In the Test cohort (similar to the Discovery cohort results), GM fr provided very good group discrimination having an AUC = 0.870. AngleR obtained the best between‐group discrimination, having an AUC = 0.931, followed by PerpPD (AUC = 0.903) and MD (AUC = 0.858). The AngleR cut‐off point determined that the “best” point of the ROC curve was 0.983 *θ*rad.

For AngleR, the likelihood ratio values (LR− = 0.15; LR+ = 20.5) revealed that individuals with AngleR values greater than 0.983 *θ*rad (Figure 3) had an increased probability of disease compared to individuals with lower AngleR values. By using an AngleR threshold of 0.983 *θ*rad, all individuals were classified with an accuracy of 90%, J 0.815, NPV (85%), and PPV (96%).

Finally, we estimated the discrimination power for each measure in the ATN (ADNI 3) cohort. This analysis confirmed the results of the “Test” and “Discovery” cohorts, showing a good discrimination power of GM fr, with an AUC = 0.798. Among diffusion cortical values, the analysis revealed that AngleR gave the best between‐group discrimination, having an AUC = 0.896, followed by PerpPD (AUC = 0.666) and MD (AUC = 0.629). AngleR had likelihood ratio values of (LR‐ = 0.05; LR+ = 3.4) revealing that individuals with AngleR values greater than 0.968 *θ*rad (Figure 3) had an increased probability of disease compared to individuals with lower AngleR values. Using a threshold for AngleR of 0.968 *θ*rad, all individuals were classified with an accuracy of 84% and a J of 0.68. This strong classification power of AngleR was also confirmed by NPV (95%) and PPV (77%).

## 4 CONCLUSION

Previous histological studies have revealed that AD results in progressive damage to minicolumn organization (Chance et al., 2011; van Veluw et al., 2012). This process, led by neurite loss and then neuronal death, causes progressive damage to the normal organization of cortical cells in columns, producing an alteration of cortical geometric properties (Chance et al., 2011; van Veluw et al., 2012). Therefore, we considered the alteration of such geometric properties as a biomarker, potentially measurable using a tailored, novel DTI analysis method. Although DTI is a relatively crude tool for analyzing diffusion MRI, it can be useful for exploring markers of disease and has been shown to relate to the underling cytoarchitecture (McKavanagh et al., 2019).

With respect to the main aims of the study, the results suggested that the cortical diffusivity analysis did detect group differences accurately and satisfy validity requirements overall.

The validity of a test is based on its ability to measure reliably (Repeatability and Reliability) for the group of variables that it is designed to measure (Construct Validity) and to correctly distinguish subjects with the disease from healthy subjects (Diagnostic Accuracy) in accordance with other pre‐existing scores (Concurrent validity). The validity of the method was tested here, to determine the possibility of generalizing the results obtained, by investigating repeatability, reliability, construct validity, and concurrent validity.

The intrascanner variation showed that the scores obtained at the two timepoints were strongly correlated and significantly consistent, with the two timepoints being much closer to each other compared to the difference between subjects, indicating a good repeatability. It must be acknowledged that there was a gap of a few weeks between time points.

The interscanner variation, assessed by applying the cortical diffusivity analysis to images from groups of healthy and AD subjects acquired on different scanners, revealed a good reliability of the cortical diffusivity analysis measures. The results obtained in all cohorts showed that subjects with AD had significantly higher values for AngleR measurements suggesting that the results were not related to the characteristics of a single sample, scanner, or operator. Further evidence from application to data across scanners came from the multicentre ATN cohort, which was an open source image dataset (ADNI 3). Although the images were from different scanners, the novel diffusion measures (in particular AngleR), remained sensitive to group differences, whereas other measures appeared susceptible to interscanner differences.

Interscanner variability represents a significant challenge in diffusion imaging. Previous studies have shown that diffusion measures can be influenced by many factors, such as acquisition protocols, analysis approaches, *b*‐value, signal‐to‐noise ratio, image resolution, scanner model, co‐registration methods, and reslicing (Bisdas, Bohning, Bešenski, Nicholas, & Rumboldt, 2008; Correia, Carpenter, & Williams, 2009; Papinutto, Maule, & Jovicich, 2013; Takao, Hayashi, Kabasawa, & Ohtomo, 2012; Zhu et al., 2011). In order to limit the number of sources of confounding variation, the present study controlled for some differences by using scans acquired with comparable protocols on scanners from the same manufacturer (Siemens). To further generalize the diffusion analysis approach presented here, additional investigations combined with harmonization methods are recommended.

The cortical diffusion analysis appeared to have good Construct Validity (Table 3) as confirmed by significant correlations between the novel diffusion measures and other commonly used measures of neurodegeneration (e.g., MMSE, Hipp bil fr, and GM_fr). AngleR values were also correlated with AV45 PET values, while PerpPD values were correlated with AV45 and AV1451 PET values. These results suggested a good relationship between the cortical diffusivity analysis measures and pre‐existing measures (Concurrent Validity), and a consistency among AD biomarkers.

The potential diagnostic accuracy of the McKavanagh et al. Method was tested using ROC curve analysis and by calculating various predictive indices (PPV, NPV, J, LR+, and LR−) estimating discriminative power. Taken together, the results showed that AngleR had the highest AUC and Accuracy among the measures considered in the analyses.

In addition, by comparing the Accuracy values for each measure in the ATN cohort, with AV45 and AV1451 PET, the discriminative power of AngleR was able to classify patients at a level similar to several conventional measures that are widely used in clinical practice (Jack Jr et al., 2018).

The structural MRI results are consistent with previous studies (e.g., Cuingnet et al., 2011), but the amyloid PET AUC differed slightly from some other studies (e.g., Palmqvist et al., 2015). Variation in PET amyloid results across studies could be due to sample size differences and/or selection of target regions. Both factors can produce significant changes in AUCs. The present study used the main whole brain amyloid value provided in the ADNI dataset.

There is, potentially, additive value in using a range of methods that provide complementary information and can provide increasing confidence of patient classification.

It is worth noting that these results are based on the diagnosis of moderate–severe AD, in order to explore the discriminatory power of the method on a well‐characterized sample with clear diagnostic indices available. This enabled evaluation of the concurrent validity of the method. Of course, the ultimate goal is to move beyond the detection of moderate–severe AD, which offers limited insight for clinical practice, toward a preliminary validation of a method that could enhance quantification of the “N” component of the ATN framework (Jack Jr et al., 2018) earlier in the disease. This could have applications in differential diagnosis (Torso, Ahmed, et al., 2020; Torso, Bozzali, et al., 2020)) with respect to other forms of dementia and ideally in early diagnosis for detecting early changes in cortical architecture. As shown by previous studies (Dubois et al., 2016), the predictive power of conventional biomarkers in the preclinical AD population requires improvement, creating a need for new biomarkers and instruments capable of more effectively detecting preclinical AD.

The objective of the ATN criteria is to separate the biomarker profile of the disease that represents the underlying pathology from the clinical diagnosis of symptoms, which can often be mimicked in other forms of disease. This raises the prospect of a potential disconnect between the biomarker and the clinical definitions and evidence of this can be seen in Figure 3, where there are a number of HC individuals who may be in the preclinical stage of AD. It is also possible that “AD” individuals who are amyloid negative should be considered atypical. Nonetheless, the findings of the present study are broadly supportive of the principle of ATN criteria.

## 5 LIMITATIONS

This study has some limitations: the study included a relatively small number in each cohort for the purpose of validation, further studies with larger cohorts would be recommended to fully generalize the findings. An additional limitation concerned interscanner reliability. In an ideal study the same individual subjects would be scanned using different acquisitions and on different scanners. This is very difficult to realize in practice, especially for subjects with AD, where a repeated acquisition would be very taxing and stressful for the patient, difficult to justify from an ethical point of view, and challenging for recruitment. All the data in the present study were drawn from existing datasets and in that respect at least, they do not represent a cohort specially optimized for our analysis.

In summary, the present study attempts to step towards building a bridge between previously characterized histopathological markers of dementia and current MRI methods. Further investigation on additional datasets will be needed, but this cortical DTI measurement, in addition to other methods already in use, appears to have the potential to contribute to improving diagnostic classification for Alzheimer's Disease. Such methods could form part of a repertoire of assessments to assist early diagnosis of the disease and differential diagnosis from other forms of dementia.

## CONFLICT OF INTEREST

The authors declare no potential conflict of interest.

## AUTHOR CONTRIBUTIONS

**Mario Torso:** Design and conceptualized study; analyzed the data; drafted the manuscript for intellectual content. **Marco Bozzali:** Major role in the acquisition of data; revised the manuscript for intellectual content. **Giovanna Zamboni:** Major role in the acquisition of data; revised the manuscript for intellectual content. **Mark Jenkinson:** Design and conceptualized study; Interpreted the data; revised the manuscript for intellectual content. **Steven A. Chance:** Design and conceptualized study; Interpreted the data; revised the manuscript for intellectual content. **Alzheimer**'**s Disease Neuroimaging Initiative (ADNI):** Acquisition of data.

## Supporting information


**Appendix** **S1**: Supporting information

**Figure S1** The cortical diffusivity mask of grey matter (see the text for more details). The color bar indicates the intensity of AngleR for each voxel.

## Data Availability

The data that support the findings of this study are available from the corresponding author upon reasonable request.
