# Supplementary material for: Detection of Alzheimer's Disease using cortical diffusion tensor imaging
Source: Hum Brain Mapp. 2020 Nov 11;42(4):967–77. doi: 10.1002/hbm.25271 (PMC7856641; doi:10.1002/hbm.25271)
Supplement: Supplementary file 1 — Appendix S1: Supporting information [file HBM-42-967-s001.docx]

**APPENDIX**

**1 MRI data acquisition**

Scanning of all subjects of the “Discovery” cohort entered in the study, was performed at the Oxford Centre for Clinical Magnetic Resonance Research using a 3T Trio Siemens MRI scanner equipped with a 12-channel head coil. The neuroimaging protocol included: (1) diffusion weighted image (DWI) acquisition SE-EPI sequence, (TR/TE = 9300/94 ms; isotropic resolution = 2.2 mm^3^; flip angle 90°; b factor = 1000s/mm^2^); this sequence collects 2 images with no diffusion weighting (b0) and 60 images with diffusion gradients applied in 60 non-collinear directions, and (2) high-resolution T1-weighted 3D MP-RAGE images (TR/TE = 2040/4.7 ms; resolution = 1 × 1 × 1 mm^3^; FOV 192x192 mm^2^).

Scanning of all subjects of the “Test” cohort was performed at the Neuroimaging Laboratory of Santa Lucia Foundation in Rome using a 3T Magnetom Allegra, Siemens MRI scanner, including the following acquisitions: (1) diffusion-weighted (DW) twice-refocused spin echo echo-planar imaging (TR/TE=10200/85 ms, isotropic resolution=2.3 mm^3^, flip angle 90°, b factor=1000s/mm^2^); this sequence collects 7 images with no diffusion weighting (b0) and 61 images with diffusion gradients applied in 61 non-collinear directions.

2) high-resolution T1-weighted 3D MDEFT (TR/TE=1338/2.4 ms; resolution = 1 × 1 × 1 mm^3^; FOV=250x250 mm^2^).

For the “ATN” (ADNI 3) cohort, MR images were acquired on 3T Siemens Prisma, Skyra and Verio scanners, using the following acquisition: (1) diffusion weighted image (DWI) (TR/TE = 7200/56 ms; isotropic resolution = 2.0 mm^3^; flip angle 90°; b factor = 1000s/mm^2^); this sequence collects 1 image with no diffusion weighting (b0) and 54 images with diffusion gradients applied in 54 non-collinear directions. (2) Accelerated Sagittal MPRAGE (TR/TE=2300/2.95 ms; resolution = 1 × 1 × 1 mm^3^; FOV=208x240x256mm).

**2 Structural MRI Analyses**

The 3D T1-weighted image for each subject was segmented using FreeSurfer v 6.0 (http://surfer.nmr.mgh.harvard.edu). This provided outputs containing estimates of cortical grey matter (GM), hippocampal (Hipp) and white matter hyperintensity (WMH) volumes. The two hippocampal volumes obtained (left and right) were averaged (Bil Hip). To account for subjects' head size differences, all volumes were expressed as a percentage of the total intracranial volume (ICV), computing GM fraction (GM fr), Bilateral Hippocampal fraction (Hipp Bil fr), and WMH fraction (WMHs fr).

**3 Cortical Diffusivity analysis**

The automatic cortical diffusivity analysis consisted of the following main stages:

**3.1 FreeSurfer segmentation**

The recon-all script, included in FreeSurfer software package 6.0 (<http://surfer.nmr.mgh.harvard.edu/>), was used to generate a segmentation of the cortex, subcortex, and white matter, create surfaces from the segmented data, and output spherical or flattened representations of these surfaces. The FreeView tool was used to visually inspect the recon-all outputs by two trained independent operators, to check the accuracy of the skull stripping and intensity normalisation procedure, the boundary between the WM and GM. Any cases where the MRI segmentation was considered to contain errors were excluded. No manual editing was performed, to avoid introducing subjective bias or increasing variance in the MRI data.

**3.2 DTI preprocessing**

DTI preprocessing was performed using FSL tools (FMRIB Software Library, Oxford, UK-http://www.fmrib.ox.ac.uk/fsl/). For each subject, the diffusion data were visually checked by two investigators (MT and SC) to detect artifacts and corrupted volumes. DTI scans of low quality were removed.

Diffusion weighted images were then corrected for motion and eddy current effects by alignment of all images to a reference b0 image using FSL’s eddy tool. The diffusion tensor was then calculated with the FSL DTIFIT tool, providing MD and V1 maps.

**3.3 EPI_reg**

The FSL epi_reg script was used to register the output of eddy (created during DTI pre-processing to achieve distortion correction) to the T1 image (after it was skull stripped). This script performs a white-matter segmentation of the structural image to define a white-matter boundary, and then uses the BBR cost function to register EPI data (diffusion image) using these boundaries (Greve & Fischl, 2009).

**3.4 Value extraction**

Novel software scripts (M.K., University of Oxford, 2018; patent application WO2016162682A1; U.S. patent application no. 15/564344) were used to generate cortical profiles on the MRI scans, that is, lines within the cortex in a radial direction, estimating the columnar organisation within the cortex. The derived measurements (AngleR, PerpPD, ParlPD) were used for analysis in the present study as whole brain average values. AngleR was calculated as the difference between the radial minicolumn direction in the cortex and the principal diffusion vector. PerpPD and ParlPD were calculated by projecting the diffusion tensor component along the principal vector on to the planes perpendicular and parallel to the radial minicolumn direction in the cortex (see also McKavanagh et al. 2019). They can also be extracted and mapped on the structural scan as shown in Supplementary Figure 1, providing more granular regional information that could be used in future studies.

The method was validated on post-mortem scans (McKavanagh et al. 2019) and tested for the first time on in-vivo AD cohorts in the present study.

**3.5 Displacement check**

To control for the effect of head motion (Baum et al. 2018) in DTI maps, a displacement index generated using an in-house script was calculated. This index measured the absolute displacement of the head from one volume to the next and was calculated as the average of the absolute values of the differentiated realignment estimates obtained from eddy correction. This value was used as a covariate in the GLM multivariate analysis.

**3.6 Erosion check**

In order to reduce any partial volume effect in the grey matter mask segmented by FreeSurfer (version 6.0) and used to estimate our cortical measures, we created 4 versions of the grey matter masks using different degrees of erosion: uneroded, and eroded by 0.5mm, 1mm and 1.5 mm. For each version, we also calculated the number of voxels. The 0.5mm mask was preferred as the best combination between reducing the partial volume effect and maintaining a sufficient number of voxels. The visual quality check revealed that all non-brain tissue and white matter were removed by the erosion.

**4 AV45 and AV1451 PET**

The most recent versions of the UC Berkeley - AV45 Analysis [ADNI1,GO,2,3] and UC Berkeley - AV1451 Analysis [ADNI1,GO,2,3] spreadsheets available at the moment of the analyses (mid 2019), were used to carry out analyses using PET values.

**5 Predictive Validity and diagnostic accuracy**

To assess the group discrimination capability for each cortical diffusivity analysis measure, we used a Receiver Operating Characteristics (ROC) curve analysis, performed with IBM SPSS Statistics version 25 (SPSS, Chicago, IL). The curve is one that plots the true positive rate against the false positive rate as the threshold is varied. The area under the curve (AUC) provides a measure of the discriminative power of a test. Comparing areas under the ROC curves we estimated which one of our cortical DTI measures is more suitable for distinguishing healthy controls from AD patients. We considered that the feature with the highest AUC (area under the ROC curve) was the best discriminator. Finally, to summarize the predictive value of each measurement, we also computed accuracy, positive likelihood ratios (LR+), negative likelihood ratios (LR-), Youden's J statistic, positive predictive value (PPV) and negative predictive value (NPV) at the best point along the ROC curve for each measurement. We defined the best value as the one with the best combination of specificity and sensitivity averaging sensitivity and 1- specificity.

Sensitivity was used to assess the proportion of true positive subjects (TP), where the disease was detected, out of the total group of subjects that had the disease, which includes the subjects considered as false negatives (FN). Sensitivity is related to the potential of a test to recognize subjects with the disease and was calculated according to the following formula:

Sensitivity = TP/(TP+FN)

Specificity was used as a measure of a diagnostic test accuracy, complementary to sensitivity, defined as a proportion of subjects without the disease and with negative test result (TN) out of the total of subjects without disease, which includes subjects without disease but with a positive test result (FP).

Specificity was calculated according to the following formula:

Specificity = TN/(TN+FP)

Accuracy is a measure of the degree of veracity of a diagnostic test on a condition, based on the proportion of true results, either true positive or true negative, out of the whole sample population.

Accuracy was calculated according to the following formula:

Accuracy = (TN + TP)/(TN+TP+FN+FP)

In addition, we used the likelihood ratio because this measure combines sensitivity and specificity and provides a summary of how much more or less likely a patient with the disease is to have a given test result (i.e., positive or negative) relative to patients without the disease (Akobeng, 2007).

The positive likelihood ratio (LR+) was computed according to the following formula:

LR+ = sensitivity/[1 – specificity]

while the negative likelihood ratio (LR-) was calculated according to the following formula:

LR- = [1 – sensitivity]/specificity

These indices are useful to translate the population characteristics (i.e., sensitivity and specificity) to single patients (Wales, 2003).

In general, large LR+ (> 10) and small LR- (< 0.10) reveals a significantly increased probability to have a target disease or virtually rule out the chance the patient has the disease, respectively (Akobeng, 2007).

Sensitivity and specificity parameters are intrinsic characteristics of the test and they depend on the type of testing adopted. They provide information about the probability of recruiting patients or healthy subjects from a certain starting population, but do not provide information about the probability that every person who tested positive really has the disease.

In the clinical epidemiology, tests are used for diagnostic purposes and not just for screening purposes. Therefore it is necessary to calculate two additional parameters: the positive and negative predictive value. Positive predictive value (PPV) is the proportion of patients with a positive test result who actually have the disease. It was computed according to the following formula:

PPV = TP / (TP + FP)

Negative predictive value (NPV) is the proportion of patients with a negative test result who do not have the disease. It was computed according to the following formula:

NPV = TN / (FN + TN)

Finally, we used Youden's J statistic (J) to estimate diagnostic accuracy and as a global measure of a test performance (Youden, 1950).

This index was calculated according to the following formula:

J = [sensitivity + specificity] – 1

J equals 0 indicates a test with poor diagnostic accuracy, while J equals 1 indicates a perfect test.

**References**

1 Akobeng, A. K. (2007). Understanding diagnostic tests 2: likelihood ratios, pre‐and post‐test probabilities and their use in clinical practice. *Acta paediatrica*, *96*(4), 487-491.

2 Greve, D. N., & Fischl, B. (2009). Accurate and robust brain image alignment using boundary-based registration. *Neuroimage*, *48*(1), 63-72.

3 Wales, N. S. (2003). Moving beyond sensitivity and specificity: using likelihood ratios to help interpret diagnostic tests. *Australian prescriber*, *26*(5).22 Youden WJ. Index for rating diagnostic tests. Cancer, 1950;3:32-35.

4 Youden, W. J. (1950). Index for rating diagnostic tests. Cancer, 3, 32–35.

**Figure 1 Cortical diffusivity mask**

Figure 1 shows the cortical diffusivity mask of grey matter (see the text for more details). The color bar indicates the intensity of AngleR for each voxel.
