# Supplementary figures and images for: Detection of Alzheimer's Disease using cortical diffusion tensor imaging
Source: Hum Brain Mapp. 2020 Nov 11;42(4):967–77. doi: 10.1002/hbm.25271 (PMC7856641; doi:10.1002/hbm.25271)

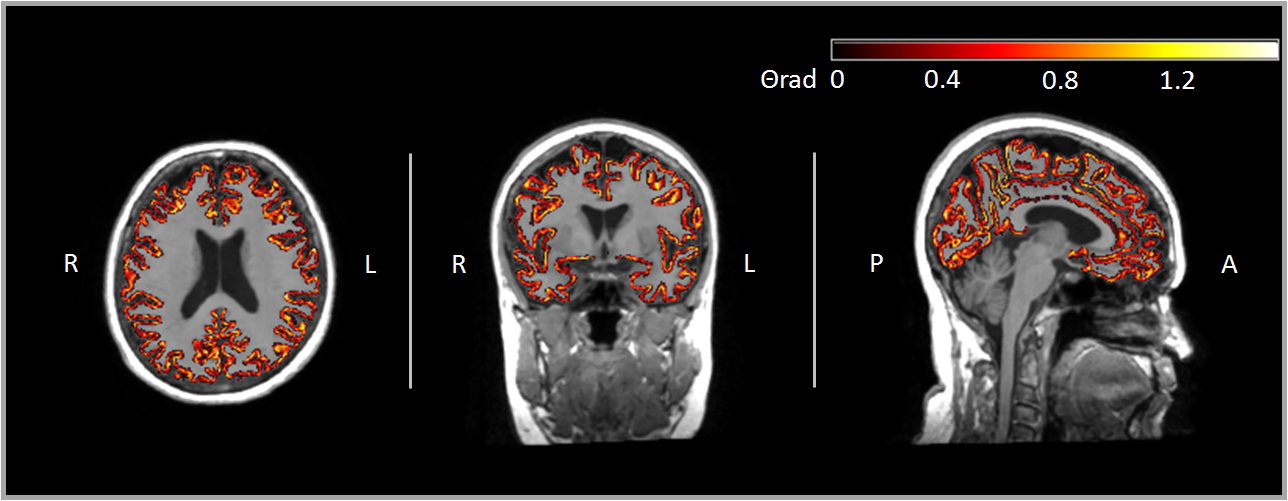

Supplement: Supplementary file 2 — Figure S1 The cortical diffusivity mask of grey matter (see the text for more details). The color bar indicates the intensity of AngleR for each voxel. [file HBM-42-967-s002.tif]
